# Artificial Intelligence in Assessing Reproductive Aging: Role of Mitochondria, Oxidative Stress, and Telomere Biology

**DOI:** 10.3390/diagnostics15162075

**Published:** 2025-08-19

**Authors:** Efthalia Moustakli, Themos Grigoriadis, Sofoklis Stavros, Anastasios Potiris, Athanasios Zikopoulos, Angeliki Gerede, Ioannis Tsimpoukis, Charikleia Papageorgiou, Konstantinos Louis, Ekaterini Domali

**Affiliations:** 1 Laboratory of Medical Genetics, Faculty of Medicine, School of Health Sciences, University of Ioannina, 45110 Ioannina, Greece; ef.moustakli@uoi.gr; 2 First Department of Obstetrics and Gynecology, Alexandra Hospital, Medical School, National and Kapodistrian University of Athens, 11528 Athens, Greecekdomali@yahoo.fr (E.D.); 3 Third Department of Obstetrics and Gynecology, University General Hospital “ATTIKON”, Medical School, National and Kapodistrian University of Athens, 12462 Athens, Greece; apotiris@med.uoa.gr (A.P.); thanzik92@gmail.com (A.Z.); giannistsimpoukis94@gmail.com (I.T.); charikleiapap@yahoo.gr (C.P.); kostaslouisss@gmail.com (K.L.); 4 Department of Obstetrics and Gynecology, Democritus University of Thrace, 69100 Campus, Greece; agerede@otenet.gr

**Keywords:** artificial intelligence (AI), reproductive aging, oxidative stress, mitochondria, telomere biology, machine learning, reproductive medicine

## Abstract

Fertility potential ever more diminishes due to the complex, multifactorial, and still not entirely clarified process of reproductive aging in women and men. Gamete quality and reproductive lifespan are compromised by biologic factors like mitochondrial dysfunction, increased oxidative stress (OS), and incremental telomere shortening. Clinically confirmed biomarkers, including follicle-stimulating hormone (FSH) and anti-Müllerian hormone (AMH), are used to estimate ovarian reserve and reproductive status, but these markers have limited predictive validity and an incomplete representation of the complexity of reproductive age. Recent advances in artificial intelligence (AI) have the capacity to address the integration and interpretation of disparate and complex sets of data, like imaging, molecular, and clinical, for consideration. AI methodologies that improve the accuracy of reproductive outcome predictions and permit the construction of personalized treatment programs are machine learning (ML) and deep learning. To promote fertility evaluations, here, as part of its critical discussion, the roles of mitochondria, OS, and telomere biology as latter-day biomarkers of reproductive aging are presented. We also address the current status of AI applications in reproductive medicine, promises for the future, and applications involving embryo selection, multi-omics set integration, and estimation of reproductive age. Finally, to ensure that AI technology is used ethically and responsibly for reproductive care, model explainability, heterogeneity of data, and other ethical issues remain as residual concerns.

## 1. Introduction

Reproductive aging of females and males is an intricate, multifactorial process that has significant effects on fertility, outcomes of pregnancy, and health of the offspring [1,2]. It is described in females as decreased quantity and quality of oocytes resulting in diminished ovarian reserve (DOR), high aneuploidy rates, and decreased reproductive capability. It is described in males as alterations in the quality of the sperm, including oxidative injury, DNA fragmentation, and alterations of telomere length [3]. The epidemiological tendency of postponed birth has widened the medical relevance of reproductive aging, placing great priority on precise diagnosis techniques for the estimation of reproductive lifespan as well as tailoring treatment regimens.

Conventional markers, including antral follicle count (AFC), FSH, and AMH exhibit limited predictive accuracy for reproductive outcomes, although they provide valuable insights into ovarian reserve [4,5]. Conversely, conventional semen analysis in males primarily assesses morphology and motility, often overlooking the underlying cellular and molecular alterations associated with reproductive aging. The quality of gametes and embryos is largely determined by three interrelated biological processes, mitochondrial dysfunction, oxidative stress (OS), and telomere biology. Recent research in reproductive biology has identified these processes as key contributors to reproductive decline and poorer reproductive outcomes. Telomere shortening functions as a cellular aging clock that restricts reproductive longevity, OS degrades DNA, proteins, and lipids necessary for gamete function, and mitochondrial impairment lowers ATP synthesis and increases reactive oxygen species (ROS) generation. Compared to traditional clinical indicators, these events collectively offer a more thorough biological basis for reproductive aging [6]. Despite the promise for these indicators to improve the assessment of reproductive aging, their integration into clinical practice is limited by the complexity and diversity of the information they provide [7,8].

AI, particularly ML and deep learning techniques, offers effective and efficient remedies to these challenges. Such technologies can process and integrate molecular profiles, clinical measures, imaging variables, and other varied information to develop forecasting systems that are more precise and robust than conventional statistical approaches [9]. Applications of AI in reproductive medicine are expanding quickly, ranging from sperm quality analysis to ovarian reserve prediction and IVF embryo selection. However, there is still little research on how artificial intelligence might be used to analyze and correlate biomarkers of reproductive aging, such as mitochondrial competence, OS measurements, and telomere dynamics [10].

We discuss the current knowledge of telomere biology, mitochondria, and OS as important indicators of reproductive aging. Subsequently, the application of AI in reproductive medicine is explored, with an emphasis on how these technologies may revolutionize the diagnosis and management of reproductive aging. The challenges of algorithm interpretability, data heterogeneity, and ethical issues in the use of AI-driven solutions are finally covered.

## 2. Mitochondrial Dysfunction in Reproductive Aging

### 2.1. The Central Role of Mitochondria in Reproductive Aging

Cells generate energy through oxidative phosphorylation (OXPHOS), which is controlled by vital organelles called mitochondria. Gamete mitochondrial activity is particularly crucial given the high energy demands of oocyte maturation, fertilization, and early embryonic development [11,12]. Oocyte mitochondria provide adenosine triphosphate (ATP) and regulate calcium homeostasis, apoptosis, and redox balance, which are critical for maintaining developmental competence and genetic stability. Sperm mitochondrial activity maintains motility and the integrity of the paternal DNA [13]. Even within the same individual throughout time, it is crucial to understand that every gamete cohort—whether from males or females—represents a distinct metabolic and functional profile. Due to this variability, a significant therapeutic window exists during which couples still producing gametes can benefit from treatments that enhance OXPHOS, improve mitochondrial function, and reduce OS. The function, quality, and viability of gametes in the context of reproductive age may be enhanced by focusing on these pathways, which could increase fertility potential and enhance the results of ART.

Reduced fertility is a result of reproductive aging-related decreases in mitochondrial quantity and quality. The accumulation of mitochondrial DNA (mtDNA) mutations, a decrease in mitochondrial membrane potential, and reduced ATP production are hallmarks of aging oocytes. These changes impair meiotic spindle formation, increasing aneuploidy rates and decreasing embryo quality. In a similar vein, elderly sperm with mitochondrial malfunction exhibit decreased motility and greater DNA breakage [14,15].

There are several methods for directly measuring mitochondrial activity in gametes. Transmission electron microscopy (TEM) to examine morphology, ATP assays for energy production, and JC-1 or TMRE labeling to determine mitochondrial membrane potential are frequently used to analyze mitochondrial activity in sperm. ATP quantification, ROS imaging to identify OS, and TEM or confocal imaging to analyze structure and distribution are used to evaluate oocyte mitochondrial function [16]. ATP measurements and oxygen consumption rate (respirometry) are utilized to assess mitochondrial function in granulosa and cumulus cells. These assessments provide essential insights into gamete quality, fertilization potential, and early embryo developmental competence [17].

#### Gamete-Specific Impacts of Mitochondrial Dysfunction

The mitochondria in the midpiece are crucial for spermatozoa to produce the ATP required for motility and capacitation. The mitochondrial membrane potential (MMP) is decreased by aging-related mitochondrial dysfunction, though, and this results in impaired motility, a reduced acrosome reaction, and increased DNA fragmentation because of the overproduction of ROS. In sperm, abnormal mitochondrial morphology and compromised mtDNA integrity have been linked to poor embryo development and decreased fertilization ability.

Granulosa and cumulus cells’ mitochondria are essential for oocyte maturation, antioxidant defense, and steroidogenesis in ovarian follicles. Functional follicular mitochondria reduce the oocyte’s developmental capacity by affecting its metabolic and redox environment. Mitochondrial DNA alterations in follicular cells have been linked to decreased ovarian reserve, whereas decreased ATP generation in cumulus cells can disrupt nutrition and signal exchange with the contained oocyte.

The energy requirements of oocytes, which have the most mitochondria in the body, peak during meiotic spindle construction and fertilization. A decrease in oocyte mitochondrial quality with age raises the probability of aneuploidy, impairs spindle formation, and lowers ATP availability. Furthermore, the development of the embryo is further jeopardized by the accumulation of mtDNA mutations and structural damage.

### 2.2. Mitochondrial Biomarkers of Reproductive Aging

An assortment of mitochondrial features has been determined to include potential indicators for assessing reproductive age. Among these, the mitochondrial DNA copy number (mtDNA-CN) has garnered significant attention [18]. Studies have indicated a relationship between mtDNA-CN in oocytes and embryos and both developmental competence and implantation capability. Reports of alterations in mtDNA-CN levels in the granulosa cells and follicular fluid of older women have raised the possibility that it could serve as a surrogate indication of oocyte quality [19].

Another possible biomarker for mitochondrial function is the mitochondrial membrane potential, or MMP. Higher apoptotic susceptibility and decreased energy production are indicated by reduced MMP in aged gametes [20]. Although JC-1 staining and other fluorometric assays have been used to measure MMP, its clinical applicability is currently primarily in the experimental stage [21].

The production of ROS is a third crucial mitochondrial characteristic. An increased ROS is mostly caused by dysfunctional mitochondria, which exacerbate OS and cause DNA damage, lipid peroxidation, and protein oxidation in gametes [22]. ROS levels in seminal plasma and follicular fluid have been frequently associated with poor reproductive outcomes, including decreased fertilization rates and embryo quality [23].

Finally, a direct measure of mitochondrial energy generation is the concentration of ATP. Aging oocytes with lower ATP levels experience challenges with cytoskeletal dynamics and meiotic spindle assembly, leading to chromosomal defects and unsuccessful fertilization. Measuring the ATP content of oocytes and embryos can provide insights into their developmental potential, even though it is technically challenging [24,25]. Table 1 summarizes the key mitochondrial biomarkers implicated in reproductive aging, including their biological roles, assessment methods, clinical relevance, and current limitations. However, these conventional methods are inherently invasive and destructive, rendering the assessed gametes unusable for ART. Recent developments in AI-driven image processing, spectroscopy, and metabolic profiling present the potential for non-invasive, point-of-care, and real-time gamete assessment. For instance, deep learning algorithms and high-resolution, label-free imaging modalities can evaluate subcellular characteristics without endangering gamete viability, potentially allowing integration into ART workflows for prompt clinical decision-making.

### 2.3. Current Challenges in Clinical Translation

Several limitations prevent mitochondrial biomarkers from becoming commonplace in the clinic, although they have good potential. One of the most significant restrictions is that there is no standard assay for the quantitative measurement of aspects like MMP and mtDNA-CN. Variation between the sensitivity of tests, treatment of samples, as well as the interpretation of results, can all cause inconsistent results between studies [30].

Another obstacle is obtaining the invasive embryo or oocyte samples required for direct analysis of the mitochondria. While less invasive, the validity of surrogate samples such as cumulus cells and follicular fluid as predictors of oocyte mitochondrial health remains under study [31]. Age, obesity, smoking, environmental contaminants, metabolic illnesses, and other confounding factors that may independently impact mitochondrial function make it more difficult to interpret biomarker data. These elements emphasize the need for advanced analytical tools capable of integrating several data dimensions while accounting for these confounders [32].

### 2.4. Potential of AI in Mitochondrial Biomarker Analysis

Given its capacity to integrate and interpret complex mitochondrial datasets, AI holds significant potential for addressing many of these challenges [33]. Through the identification of complex patterns and interactions among variables such as mtDNA-CN, ROS levels, and patient demographics, machine learning algorithms can predict oocyte quality and fertility results more precisely than traditional statistical methods [34].

Analysis of data from high-resolution mitochondrial imaging has demonstrated the effectiveness of deep learning methods, particularly convolutional neural networks (CNNs). These methods could be used to detect subtle structural or functional abnormalities in mitochondria that are not obvious to human observers [35].

Furthermore, a promising approach to thorough modeling of reproductive aging is AI-driven multi-omics integration [36]. Through the integration of proteomics, metabolomics, and mitochondrial genomes data, artificial intelligence frameworks can offer a comprehensive perspective on mitochondrial health and its correlation with reproductive capacity [37]. Personalized fertility treatment plans and non-invasive, precise prediction tools may be made possible by such methods [38,39].

## 3. OS as a Biomarker

### 3.1. Role of OS in Reproductive Aging

An imbalance between the generation of ROS and the antioxidant defense systems that counteract them results in OS [40]. Normal reproductive activities, including folliculogenesis, oocyte maturation, sperm capacitation, and embryo development, depend on physiological levels of ROS, whereas excessive ROS creation damages cells. This damage includes DNA fragmentation, protein oxidation, and lipid peroxidation, all of which are especially harmful to the reproductive system’s highly specialized cells [41,42].

OS has been linked to age-related declines in ovarian reserve and oocyte quality in women [43]. Increased ROS buildup and decreased antioxidant capability in aging ovaries are linked to meiotic errors, aneuploidy, and granulosa cell death. OS is a major factor in men’s sperm quality declining with age, leading to DNA breakage, decreased motility, and compromised fertilization ability [44].

OS in gametes can be directly assessed using a variety of techniques. While DNA fragmentation can be evaluated using TUNEL or Comet assays, ROS levels in sperm are often quantified using DCFDA labeling or chemiluminescence tests. Oxidative balance in oocytes is assessed by ROS imaging and antioxidant enzyme activity assays (e.g., SOD, catalase), and oxidative damage can also be deduced from DNA oxidation indicators or lipid peroxidation products. Similar biochemical tests are used to evaluate ROS levels and antioxidant capacity in granulosa and cumulus cells, which shed light on the follicular microenvironment and its capacity to promote oocyte development [45].

#### Gamete-Specific Impacts of OS

For functions including capacitation and hyperactivation, sperm require physiological amounts of ROS; nevertheless, too much ROS can harm sperm proteins, lipids, and DNA, decreasing motility and increasing morphological abnormalities. Male infertility, especially in older men, has been closely linked to elevated OS in seminal plasma.

Granulosa and cumulus cells’ ability to function is hampered in ovarian follicles by OS in the follicular fluid, which changes the microenvironment surrounding the oocyte. In ART cycles, lower fertilization rates and lower-quality embryos are associated with elevated lipid peroxidation products in the follicle, such as malondialdehyde (MDA).

Oocytes are particularly vulnerable to oxidative injury due to their large cyto-plasmic volume and prolonged meiotic arrest. The consequences of aging on reproductive capacity can be exacerbated by excessive ROS, which can cause spindle integrity disruption, mitochondrial malfunction, and telomere shortening.

### 3.2. OS Biomarkers in Reproductive Aging

Several biomarkers have been proposed for the evaluation of OS within the reproductive system. ROS levels in follicular fluid, seminal plasma, or cumulus cells can be directly measured to ascertain the oxidative environment surrounding gametes [46,47]. Reduced implantation success, lower fertilization rates, and poorer embryo quality have been linked to increased ROS concentrations in ART [48,49].

Indirect indicators of oxidative damage are the markers of lipid peroxidation MDA and 4-hydroxynonenal (4-HNE). These have been reported to be present in increased quantities within infertile men’s seminal plasma and women suffering from DOR’s follicular fluid [50]. Oxidation of reproductive proteins required for gametic functionality is also suggested by markers of protein oxidation, such as advanced oxidation protein products (AOPPs) [51].

Measuring antioxidant capacity with specific enzymes such as catalase, glutathione peroxidase (GPx), and superoxide dismutase (SOD) or total antioxidant capacity (TAC) can yield more details on the oxidative equilibrium. These antioxidant defenses have been shown to decline with age and infertility in both sexes [52]. Table 2 summarizes the major biomarkers of OS associated with reproductive aging. Gametes meant for ART cannot be immediately subjected to OS evaluations since the majority of them still require destructive testing or invasive sampling. By combining label-free imaging, fast spectroscopic readouts, and non-invasive metabolic profiling, advances in AI-assisted analysis provide a mechanism to evaluate oxidative status in real time at the point of treatment while maintaining gamete integrity.

### 3.3. Current Challenges in Biomarker Utilization

Despite growing evidence that OS contributes to reproductive aging, the therapeutic use of OS biomarkers remains limited [57]. The variation in assay techniques used to assess ROS and antioxidant levels is a major issue that contributes to contradictory research outcomes. The assessment of OS is complicated by its dynamic character, which varies in response to clinical states, environmental exposures, and lifestyle factors [58].

Additionally, the intrusive collection of follicular fluid or cumulus cells for OS testing limits its usual use in reproductive clinics [59]. Men’s semen collection is less intrusive, but interpretation is made more difficult by the variety of OS indicators in seminal plasma. A crucial first step in integrating biomarker assays into reproductive medicine is standardizing them and creating clinically meaningful reference ranges [60,61,62].

### 3.4. Potential of AI in OS Biomarker Analysis

By combining intricate datasets and identifying patterns that are difficult to identify using traditional techniques, artificial intelligence holds the potential to revolutionize the research of OS biomarkers [63]. To create prediction models of reproductive outcomes, ML algorithms can integrate clinical indicators, OS markers, antioxidant levels, and patient lifestyle factors [64].

Fluorescent ROS probes in gametes or embryos are examples of high-content OS imaging that deep learning techniques could examine to find minute oxidative signatures linked to aging [65]. AI frameworks can facilitate the integration of mitochondrial and telomere biomarkers with OS data, enabling a more comprehensive assessment of gamete quality and reproductive potential. Non-invasive, customized methods to track OS and improve the results of reproductive treatments may be made possible by these advancements [10].

## 4. Telomere Biology in Fertility

### 4.1. Telomere Shortening and Reproductive Lifespan

Telomeres, repetitive nucleotide sequences that cap the ends of chromosomes, function as a biological clock for cellular aging by progressively shortening with each cell division. The reduction in reproductive lifespan is closely related to this shortening [66]. Oocyte telomere attrition in females is linked to greater rates of infertility and miscarriage, as well as decreased ovarian reserve and oocyte quality. Male is adversely affected by spermatozoa with shortened telomeres, which have been linked to reduced sperm motility, increased DNA fragmentation, and lower fertilization rates [67].

Several well-established methods can be used to evaluate the integrity and length of the telomeres in gametes. While fluorescence in situ hybridization (FISH) can show telomere distribution, qPCR or terminal restriction fragment (TRF) analysis are typically used to quantify telomere length in sperm. Q-FISH is frequently used to assess oocyte telomeres because it enables the direct assessment of telomere length and the identification of dangerously short telomeres. The ability of granulosa and cumulus cells to support oocyte development and overall reproductive potential can be determined by measuring their telomere length using qPCR or FISH [68]. Current methods for measuring telomeres in gametes usually involve destructive sampling, which precludes the evaluated cells from being used in other clinical settings. Without sacrificing the viability of gametes for ART, quick, real-time telomere evaluation at the point of treatment may be possible by combining non-invasive optical or molecular profiling with AI-driven picture and data processing.

#### Gamete-Specific Telomere Dynamics

As paternal age increases, telomerase activity in spermatogonial stem cells tends to maintain or even increase telomere length in sperm. Nonetheless, telomeric DNA can still be harmed by severe OS, which can lead to fragmentation of sperm DNA and decreased embryo viability.

Follicle somatic cells in ovarian follicles, such as cumulus and granulosa cells, gradually shorten their telomeres as they age. Oocyte quality may be compromised if these supporting cells’ telomeres are shortened, which could affect their capacity to protect and nourish the oocyte.

Due to the low telomerase activity after birth, oocytes show gradual telomere attrition throughout a woman’s reproductive lifespan. Chromosome mis-segregation, meiotic spindle instability, and an elevated risk of miscarriage are all associated with oocytes with short telomeres. This attrition is further exacerbated by mitochondrial dysfunction and OS, creating a synergistic pathway that contributes to reproductive decline.

### 4.2. Male vs. Female Gametes: Telomere Dynamics

Male and female gametes have distinct telomere length dynamics as a result of differences in gametogenesis and telomerase activity. Since spermatogonial stem cells contain active telomerase, sperm telomeres often lengthen as paternal age increases [69]. However, oocytes lack considerable telomerase activity after birth, resulting in increasing telomere shortening with maternal age. This sexual dimorphism identifies distinct molecular mechanisms in gamete aging and emphasizes the significance of considering sex-specific telomere biology in reproductive research [70].

### 4.3. AI Approaches to Telomere Length Prediction

AI and ML techniques have emerged as very efficient methods for the analysis of telomere length from complex genomic and clinical data [71]. By integrating clinical traits and multi-omics data, AI models can precisely predict telomere length, enabling early detection of reproductive aging and risk stratification for infertility. These techniques may improve reproductive outcomes and allow for tailored fertility treatments by directing decisions regarding the timing and strategies of interventions [72].

## 5. AI in Reproductive Medicine

### 5.1. Current Applications of AI

AI has accelerated the transformation of reproductive medicine by enhancing clinical decision-making and diagnostic precision [73]. IVF selection is streamlined by embryo grading systems that assess embryo morphology and developmental potential using deep learning algorithms and sophisticated image analysis [74]. Furthermore, by examining patient clinical data and treatment parameters, prediction models have been created to forecast IVF success rates. AI-driven sperm quality evaluations in male fertility use pattern recognition and computer vision to detect sperm motility, morphology, and concentration more reliably and accurately than conventional techniques [75].

### 5.2. AI for Multi-Omics Integration

Reproductive biology is composed of complex interactions of the genome, proteasome, and metabolome. By integrating multi-omics datasets, AI techniques reveal new biomarkers and pathways involved in infertility and reproductive aging [76]. Extracting patterns and relationships between disparate sets of data that are routinely ignored by conventional analytical techniques, machine learning programs provide detailed insights into the health of gametes, embryo development, and processes of aging. Personalized reproductive health might be advanced by the integrated AI framework by customizing drugs based upon complex biological realities [77].

### 5.3. Case Studies and Existing AI Models in Reproductive Aging

Despite being a relatively recent focus, reproductive aging has prompted the development of several AI models. For example, by analyzing hormone patterns in combination with genetic markers, several studies have used machine learning to predict the decline of ovarian reserve [78]. Other models use imaging data to assess ovarian follicle quality and forecast when menopause will begin. These case studies, while still in their infancy, show how AI may advance our knowledge of and strategy for dealing with reproductive aging, opening the door for therapeutic applications that might prolong windows of fertility or maximize timing for fertility preservation [78]. Table 3 summarizes the key applications of AI in reproductive medicine.

### 5.4. AI for Integrating Complex Datasets in Reproductive Aging

**Figure 1:**
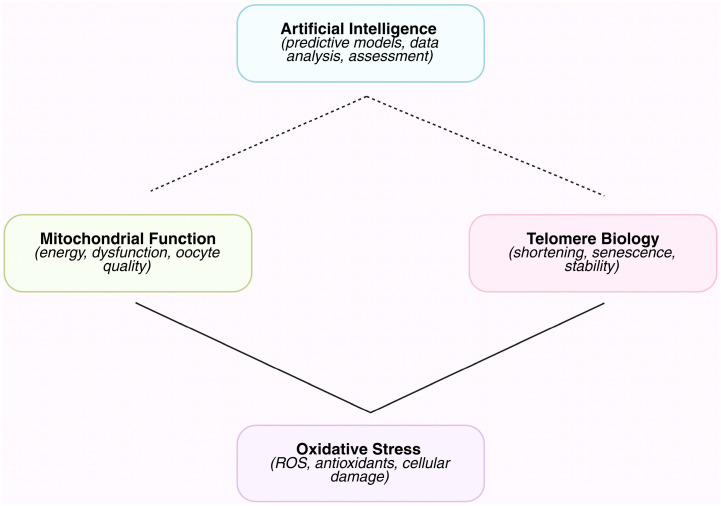
Schematic overview of the interplay between AI approaches and key mechanisms involved in reproductive aging. AI tools are applied to assess, predict, and monitor mitochondrial function, OS, and telomere biology. Solid arrows represent well-established mechanistic links, while dashed arrows indicate emerging or hypothesized connections. This schematic highlights how AI can integrate multifactorial biological data to provide insights into reproductive aging.

The biological processes involved in reproductive aging are intricately linked and include OS, telomere attrition, and mitochondrial dysfunction. These processes are measured in a variety of gametes and supporting cells. Conventional studies cannot fully represent the biological complexity of the generated datasets since they are massive, multidimensional, and frequently non-linear [82]. AI provides the computing power to merge these diverse datasets—clinical, molecular, imaging, and functional—into logical models that can identify trends linked to less successful reproductive outcomes. To predict oocyte or sperm quality, aneuploidy risk, and embryo developmental potential, for example, machine learning can combine patient-specific reproductive features with data on mitochondrial activity, ROS levels, and telomere length. AI promotes tailored approaches to slow fertility decline and allows for a more comprehensive understanding of reproductive aging by combining intricate measures into useful insights (Figure 1) [72,83].

## 6. Challenges and Future Directions

### 6.1. Data Heterogeneity and Standardization

AI applications in reproductive aging are emerging, with existing models representing only the initial stages of development. ML has been used in several studies to predict DOR by examining hormone patterns in conjunction with genetic markers. Other models utilize imaging data to predict the onset of menopause and assess the health of ovarian follicles [77]. These early-stage case studies demonstrate how AI can improve our understanding and treatment of reproductive aging. This may lead to therapeutic applications that extend windows of viability or enhance timing for fertility maintenance [38].

### 6.2. Utilization of AI Models

Even though AI models, particularly deep learning networks, have a high predictive accuracy, their “black-box” nature makes clinical implementation difficult. Understanding how models make decisions, or explainability, is necessary to win over patients’ and physicians’ trust. Incorporating transparent reasoning techniques and developing interpretable AI frameworks will enable validation, regulatory approval, and ethical application in fertility care [84].

### 6.3. Explainability of AI Models

Reproductive medicine has used a number of different machine learning techniques, each with special benefits based on the kind and complexity of the data. CNNs are especially good at evaluating imaging data, including sperm morphology evaluations and time-lapse embryo movies. Their layered architecture makes it possible to identify tiny morphological variations that could be hard for human observers to notice by automatically extracting hierarchical image attributes. A CNN trained on more than 12,000 annotated embryo photos outperformed traditional morphological grading in one case study in terms of implantation prediction accuracy.

For tabular clinical data, such as patient information, hormone levels, and genetic markers, tree-based ensemble models like Random Forest are frequently utilized. Random Forest can handle diverse datasets with both continuous and categorical variables and minimize overfitting by merging several decision trees. For instance, a Random Forest model with significant cross-clinical generalizability was trained on a multi-center dataset of 2000 IVF cycles and was able to predict clinical pregnancy rates.

This strategy is expanded by gradient boosting algorithms like XGBoost, which construct trees one after the other with an emphasis on fixing the mistakes of the earlier ones. High prediction performance, effective management of missing data, and the capacity to identify intricate non-linear relationships are all hallmarks of XGBoost. With an area under the ROC curve (AUC) above 0.85 in validation cohorts, XGBoost models have been utilized in reproductive aging research to combine genetic, hormonal, and lifestyle data to estimate the likelihood of decreased ovarian reserve.

For these models to function at their best, access to well-annotated datasets is still essential. Training material can be obtained from publicly accessible sources, such as the Human Fertilization and Embryology Authority (HFEA) database for IVF results and curated imaging libraries for embryo development. To enable solid, repeatable AI research in reproductive medicine, collaborative data-sharing activities are necessary, as many high-quality datasets are still proprietary or limited.

### 6.4. Ethical and Legal Considerations

The application of AI in reproductive medicine raises important ethical and legal concerns [85]. Ensuring fair and responsible AI use requires addressing patient privacy, data security, informed consent, and biases in training data. Regulations specific to reproductive health applications are required to protect patient rights and advance equitable access to cutting-edge AI technologies [86].

### 6.5. Personalized Fertility Medicine Powered by AI

AI might transform future fertility treatment by supplying extremely personalized drugs [38]. Multi-dimensional information, like genetic and behavioral character traits, might be combined by AI to maximize therapy timing, personalize therapies based on each unique patient profile, and enhance results. Effective validation, ethical oversight, and continued innovation can unlock AI-led personalized fertility care [87].

### 6.6. Telomere Biology in Reproductive Aging: Measurement, Mechanisms, and Therapeutic Potential

Telomere length is increasingly recognized as an important biomarker of reproductive aging. It can be measured through different methods, each with their own advantages and disadvantages. Rapid, high-throughput analysis is possible using qPCR, although it only provides estimates of relative length. Although it allows for absolute length values, TRF analysis is less appropriate for small reproductive samples and necessitates substantial amounts of DNA. Individual gametes or embryos can be observed using Q-FISH, but more recent methods like telomere shortest length assay (TeSLA) and single telomere length analysis (STELA) improve the detection of critically short telomeres, which may be the most clinically relevant [88].

There is growing recognition that telomere dynamics differ by sex. Male sperm telomeres can be maintained or even extended over time by telomerase activity during spermatogenesis, while female oocytes incur progressive telomere attrition, which is made worse by mitochondrial malfunction and OS. With androgens affecting telomere stability through oxidative pathways and estrogen having been demonstrated to increase telomerase activity, hormonal regulation may also play a role [69].

Pharmacologic telomerase activators, antioxidant supplements, and lifestyle modifications to reduce OS are examples of potential telomere-targeted therapies that have shown some initial promise in preclinical animals. Though they are being investigated, more sophisticated strategies—like gene therapy to increase telomerase expression—need to be carefully evaluated for safety because of possible carcinogenic concerns. Using non-invasive biomarker screening to choose gametes or embryos with longer telomeres may improve the results of ART. Long-term safety and ethical issues must be weighed against these promising treatments, particularly when it comes to younger patient populations [89,90].

### 6.7. Limitations and Ethical Challenges of AI in Reproductive Medicine

Despite the promising advances in AI applications for reproductive medicine, there are important limitations that must be addressed before these technologies can be widely adopted in clinical practice. Given that AI models significantly depend on the caliber and representativeness of their training data, dataset bias is one of the main issues. Due to a lack of diversity in variables including age, ethnicity, and clinical conditions, many of the datasets currently in use may produce skewed forecasts that exacerbate healthcare inequities. For instance, when applied to underrepresented groups, models that were primarily trained on data from particular populations may perform poorly, which could lead to unequal outcomes for fertility care [91].

Model validation is another important concern. Thorough testing across several independent cohorts is necessary to determine the generalizability and dependability of AI models. However, single-center data and small sample sizes are common in reproductive medicine research, which restricts external validity. Comparing and evaluating various models is made more difficult by the lack of defined procedures for data gathering and result measurement [92].

Practical difficulties can arise when integrating AI models into clinical settings. Early-stage research occasionally ignores important steps like integration with current electronic health record systems, guaranteeing compatibility with clinical workflows, and offering sufficient user training. Furthermore, many AI algorithms are “black-box” in nature, which limits physicians’ capacity to fully understand the predictions and confidently apply them to clinical decision-making [91].

Informed consent, algorithm openness, and patient privacy are ethical issues. The utilization of their data and the creation of AI-generated suggestions may not always be clear to patients. Additionally, AI may be used in ways that compromise patient autonomy or inadvertently reinforce preexisting biases [93].

Finally, securing governmental approval, protecting data, and defining who is responsible for AI blunders are some of the obstacles to real-world application. For example, it remains unclear who would be held accountable—the healthcare facility, the AI developer, or the clinician—if an AI model forecasts embryo viability inaccurately, resulting in a failed IVF cycle. Clinical professionals, data scientists, ethicists, and regulators must work together to address these intricate issues and guarantee that AI tools be used in reproductive care in a way that is safe, efficient, and morally sound [94].

## 7. Conclusions

This review underscores the essential link between mitochondrial dysfunction, OS, telomere biology, and fertility by illustrating the ways in which these interconnected processes determine gamete quality and reproductive duration for females and males. Key mechanisms of reproductive aging and poorer reproductive outcomes can be explained by telomere shortening acting as a cellular aging clock, OS causing molecular damage that compromises fertilization and embryo development, and mitochondrial impairment decreasing energy production and increasing ROS generation. By facilitating advanced analysis of sperm, precise IVF chance prognosis, better embryo grading, and incorporation of sophisticated multi-omics data, artificial intelligence has transformed reproductive medicine. Aging can now be better understood and managed due to these advances.

Data heterogeneity, model explainability requirements, and substantial ethical and regulatory challenges are just some of the challenges for the implementation of AI technology in this industry. AI technology solutions have to overcome these challenges for them to remain trustworthy, fair, and therapeutically valuable.

AI-centered methods of the future have unprecedented potential to provide customized diagnosis, customized fertility care, and precise reproductive age determination. It would take an interdisciplinary approach and innovation in technology to realize the full potential of AI and ultimately improve fertility and reproductive health outcomes across varied groups.

## Figures and Tables

**Table 1:** Key mitochondrial biomarkers implicated in reproductive aging, including their biological roles, assessment methods, clinical relevance, and current limitations.

Biomarker	Biological Role	Assessment Method	Clinical Relevance	Limitations
mtDNA-CN [26]	Indicator of mitochondrial biogenesis	Quantitative (qPCR), digital PCR	Lower copy number is associated with reduced oocyte developmental competence and poorer embryo implantation potential	Requires invasive sampling of oocytes/embryos; inter-assay variability due to extraction and quantification differences
MMP [27]	Reflects mitochondrial functionality	JC-1, TMRE, Rhodamine dyes	High MMP correlates with better fertilization and blastocyst formation rates	Fluorescent dyes may yield inconsistent results between labs; signal intensity affected by staining time and temperature
ROS [28]	Byproduct of mitochondrial dysfunction	Chemiluminescence, fluorometric assays	Elevated ROS is linked to oxidative damage, impaired fertilization, and reduced embryo quality	Levels fluctuate rapidly in response to handling, oxygen exposure, and culture conditions; requires rapid measurement post-collection
ATP Content [29]	Direct measure of energy production	Bioluminescence assays	Adequate ATP is essential for spindle assembly, chromosome segregation, and normal fertilization	Requires destruction of the gamete/embryo for measurement; susceptible to degradation during sample processing

**Table 2:** Major biomarkers of OS relevant to reproductive aging, categorized by type (direct oxidative markers, lipid peroxidation, protein oxidation, and antioxidant status). Assessment methods, associations with gamete and embryo quality, and current challenges in clinical application are included.

Biomarker	Type	Assessment Method	Clinical Relevance	Limitations
ROS [53]	Direct oxidative marker	Fluorescence probes, EPR spectroscopy	Elevated ROS levels in gametes and reproductive fluids are linked to DNA damage, reduced fertilization rates, and poorer embryo quality in aging individuals	Highly unstable and short-lived; measurements can vary with handling time, oxygen exposure, and culture conditions
MDA [41]	Lipid peroxidation	TBARS assay, HPLC	Increased MDA in follicular fluid and seminal plasma correlates with membrane damage and reduced gamete viability in older patients	Not specific to reproductive tissues; TBARS can overestimate due to interference from other aldehydes
4-HNE [54]	Lipid peroxidation	Immunoassays, ELISA	Accumulates in oocytes during aging, contributing to spindle abnormalities and impaired embryo development	Limited clinical threshold data; cross-reactivity with similar aldehydes can affect accuracy
AOPPs [55]	Protein oxidation	Spectrophotometric assays	Elevated AOPPs in serum and follicular fluid are associated with subfertility and accelerated reproductive aging	Systemic OS can influence results; cannot differentiate between local and systemic protein oxidation sources
TAC [56]	Antioxidant status	FRAP, ABTS, ORAC assays	Reduced TAC in follicular fluid and seminal plasma correlates with diminished antioxidant defense and poor ART outcomes in older patients	Strongly influenced by recent diet, supplementation, and lifestyle factors; no universally accepted reference range for reproductive fluids

**Table 3:** Key applications of AI in reproductive medicine.

Application Area	Description	Input Types	AI Techniques	Clinical Impact
Embryo Grading [74]	Automated assessment of embryo morphology and viability	Embryo images, time-lapse videos	Deep learning, CNNs	More consistent and objective embryo selection, leading to improved implantation and live birth rates in IVF
IVF Success Prediction [79]	Predicts likelihood of successful implantation and pregnancy	Patient clinical data, hormonal profiles	ML, predictive modeling	Enables individualized treatment protocols, potentially reducing cycle numbers and costs
Sperm Quality Analysis [80]	Evaluates sperm motility, morphology, and concentration	Sperm microscopy images, semen analysis	Computer vision, pattern recognition	Enhances diagnostic accuracy and reproducibility in male infertility assessment
Multi-Omics Integration [81]	Combines genomics, proteomics, metabolomics data for fertility insights	Genomic sequences, protein expression, metabolites	ML, data integration algorithms	Discovery of novel biomarkers and personalized therapies
Reproductive Aging Prediction [78]	DOR models and menopause onset	Hormonal profiles, genetic markers, imaging data	ML, regression models	Early detection of reproductive aging, fertility preservation decisions

## Data Availability

Data sharing is not applicable to this article as no new data were created or analyzed in this study.
